# Deleted in lymphocytic leukemia 2 (DLEU2): a possible biomarker that holds promise for future diagnosis and treatment of cancer

**DOI:** 10.1007/s12094-023-03149-x

**Published:** 2023-04-24

**Authors:** Xue Qu, Yu-xia Cao, Yuan-xin Xing, Qi Liu, Huan-jie Li, Wei-hua Yang, Ban-qin Wang, Shu-yi Han, Yun-shan Wang

**Affiliations:** 1 Shandong First Medical University, No. 6699, Qingdao Road, Huaiyin District, Jinan, 250117 Shandong China; 2 Medical Research and Laboratory Diagnostic Center, Central Hospital Affiliated to Shandong First Medical University, No.105, Jiefang Road, Jinan, 250013 Shandong China; 3 Research Center of Basic Medicine, Central Hospital Affiliated to Shandong First Medical University, No.105, Jiefang Road, Jinan, 250013 Shandong China; 4 Shandong University, No. 44, Wenhua West Road, Jinan, 250100 Shandong China; 5 Medical Integration and Practice Center, Shandong University, Jinan, China; 6 Department of Blood Transfusion, the First Affiliated Hospital of Shandong First Medical University & Shandong Provincial Qianfoshan Hospital, Jinan, 250014 China

**Keywords:** lncRNA-DLEU2, Cancer, Biomarker, Molecular mechanisms, Functions

## Abstract

The mechanism of deleted in lymphocytic leukemia 2 (DLEU2)-long non-coding RNA in tumors has become a major point of interest in recent research related to the occurrence and development of a variety of tumors. Recent studies have shown that the long non-coding RNA DLEU2 (lncRNA-DLEU2) can cause abnormal gene or protein expression by acting on downstream targets in cancers. At present, most lncRNA-DLEU2 play the role of oncogenes in different tumors, which are mostly associated with tumor characteristics, such as proliferation, migration, invasion, and apoptosis. The data thus far show that because lncRNA-DLEU2 plays an important role in most tumors, targeting abnormal lncRNA-DLEU2 may be an effective treatment strategy for early diagnosis and improving the prognosis of patients. In this review, we integrated lncRNA-DLEU2 expression in tumors, its biological functions, molecular mechanisms, and the utility of DLEU2 as an effective diagnostic and prognostic marker of tumors. This study aimed to provide a potential direction for the diagnosis, prognosis, and treatment of tumors using lncRNA-DLEU2 as a biomarker and therapeutic target.

## Introduction

Today, modern medicine and technology are developing rapidly. However, cancer remains one of the main causes of human death. The latest global cancer statistics show that 19.29 million new cancer cases and 9.96 million cancer deaths occurred globally in 2020. Owing to the serious aging of the global population, it is estimated that the cancer burden will increase by 50% in 2040 compared to that in 2020, and the number of new cancer cases will reach 30 million [1]. Despite continuous advances in cancer treatment, early diagnostic markers and effective therapeutic targets are lacking, resulting in poor prognosis [2]. Therefore, the development of new biomarkers and therapeutic targets for early diagnosis can greatly reduce cancer-related mortality and morbidity.

Long non-coding RNAs (lncRNAs) are non-coding RNAs (ncRNAs) with lengths greater than 200 nts. Although lncRNAs do not encode proteins, they perform complex biological functions [3]. In recent years, this has become a popular research topic. Previous research has found that nuclear lncRNAs interact with proteins, DNA, RNA, or other biological molecules; cytoplasmic lncRNAs inhibit mRNA by directly targeting mRNA or by competitive endogenous RNA (ceRNA) mode, which regulates tumor proliferation, mutation, migration, cell cycle, invasion, and metastasis processes, and may be considered as a new potential prognostic marker and future biomarkers and targets for cancer treatment [4]. An increasing number of studies have shown that mutations and abnormal lncRNA expression can lead to multiple diseases, including cancer [5].

DLEU2 is an lncRNA, and its host gene is located at site 49,982,552–50,125,541 on chromosome 13 with 15 exons. Abnormal expression of this gene locus frequently occurs in leukemia [6] and solid tumors [7], and is a popular research topic in tumor research. This gene was initially identified as a potential tumor suppressor in B cell chronic lymphocytic leukemia (B-CLL) [6], and in subsequent studies, it was found DLEU2 gene was also significantly downregulated in pediatric acute myeloid leukemia (AML) [8]. DLEU2 is an emerging lncRNA in recent years, and its expression is upregulated in a variety of tumors and is highly correlated with clinical prognosis [7], suggesting that lncRNA-DLEU2 (hereafter referred to as DLEU2) may play a role in various tumors as a potential biomarker or future clinical therapeutic target. Here, the expression, function, molecular mechanism, and clinical application of DLEU2 in tumors will be clarified in detail to gain an in-depth understanding of the diagnosis, prognosis, and therapeutic potential of DLEU2.

## DLEU2 in cancer

**Fig.1:**
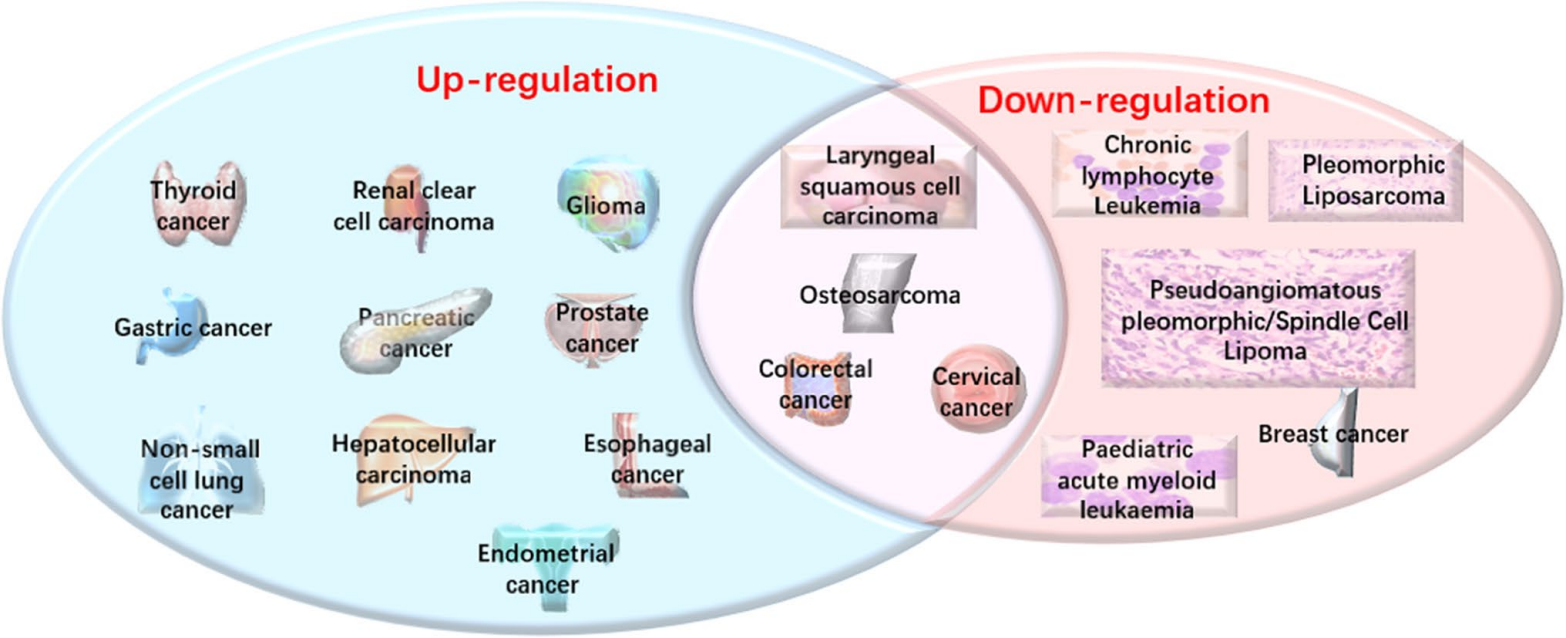
Expression of DLEU2 in different tumors

More recently, DLEU2 has been reported to be abnormally expressed in many malignant tumors. For example, DLEU2 is downregulated in chronic lymphocytic leukemia (CLL) [6], pleomorphic liposarcoma (PL) [9], Pseudoangiomatous Pleomorphic/Spindle Cell Lipoma [10], pediatric AML [8], and breast cancer (BC) [11]. However, most reports have shown upregulation of DLEU2 in cancers, including endometrial cancer(EC) [12], renal clear cell carcinoma(ccRCC) [13], esophageal cancer (ESCA) [14], pancreatic cancer (PC) [15], glioma [16], gastric cancer(GC) [17], non-small cell lung cancer (NSCLC) [18], thyroid cancer(TC) [19], and prostate cancer (PCa) [20]. These studies suggest that DLEU2 exhibits different functions in different cancers because of its tissue specificity [21]. Notably, DLEU2 expression also differed within the same cancer. Studies have confirmed that the low expression of DLEU2 in laryngeal cancer (LC) tissues is in sharp contrast to that in normal tissues [22]. However, this effect is reversed in laryngeal squamous cell carcinoma (LSCC). Compared to that in normal tissues, the expression level of DLEU2 in LSCC is significantly increased [23]. In addition, DLEU2 expression is different in cervical cancer (CC) [21, 24], osteosarcoma [25, 26], and colorectal cancer (CRC) in the same cancer [27, 28] (Fig. 1). Such inconsistencies may be attributed to differences in cancer subtypes, tumor heterogeneity, or transcripts (Table 1). Different transcripts may lead to different biological functions or target different molecules and signaling pathways. Further research is required to make DLEU2 more precise and accurate in clinical treatment. In addition, abnormal expression of DLEU2 is closely related to the clinicopathological features and poor prognosis of patients. This section critically discusses the expression and clinical significance of DLEU2 in tumors.

**Table 1:** DLEU2 transcripts in different tumors

Transcript variant	Cancer type	Expression status (up/down)	Function	Type of cell line	Targets	References
1/2/5/6/7	Endometrial cancer (EC)	Up	EMT, Glycolysis Invasion Migration Chemoresistance Sphere formation	HEC-1 HEC-50 Ishikawa, KLE HHUA, EM	EZH2 miR-181a miR-455, HK2 p-FAK ERK1/2	[12]
	Hepatocellular carcinoma (HCC)	Up	–	Huh-7, SNU449 MIHA	–	[39]
			Proliferation Invasion Migration	LO-2, Huh-7 SNU1 HCCLM3 SK-HEP-1 SMMC7721	EZH2	[30]
	Thyroid cancer (TC)	Up	Proliferation Migration Glycolysis Apoptosis	Nthy-ori 3-1 TPC-1 BHT-101	miR-205-5p TNFAIP8	[19]
	Glioma	Up	Proliferation Invasion Migration	NHA, U87 U251, U343 A172, LN229	miR-186-5p PDK3	[16]
	Osteosarcoma	Down	Invasion Migration	MG-63, U-2OS hFOB1.19	miR-15a Bcl-2	[26]
	Laryngeal carcinoma (LC)	Down	Proliferation Invasion Migration	TU212, A549	miR-16-1	[22]
1	Prostate cancer (PCa)	Up	Proliferation Invasion Migration	PC-3, DU145	miR-582-5p SGK1	[20]
	Esophageal cancer (ESCA)	Up	Proliferation Invasion Migration Apoptosis	Eca-109 KYSE-150 TE-1	miR-30e-5p E2F7	[31]
	Gastric cancer (GC)	Up	Proliferation Migration EMT	MKN-45 GES1 SGC-7901 HGC-27 NCI-N87, AGS BGC-823 MGC-803	miR-30a-5p ETS2,p-AKT	[17]
	Cervical cancer (CC)	Up	Proliferation Apoptosis	HeLa, SiHa CaSKi C-33A, HaCaT	miR-128-3p	[21]
	Laryngeal squamous cell carcinoma (LSCC)	Up	Proliferation Invasion Migration	Hep2 AMP-HN-8	miR-30c-5p PIK3CD,Akt	[23]
	Non-small cell lung cancer (NSCLC)	Up	Proliferation, Invasion	A549 NCI-H460	miR-30a-5p PHTF2	[18]
2/3/4/5/6/7	Clear cell renal cell carcinoma (ccRCC)	Up	Proliferation Invasion Migration, EMT	P, 780-O ACHN Caki-1, HK2	miR-30a-5p ZEB2	[13]
	GC	Up	Proliferation Invasion Migration	HGC-27 AGS BGC-823 SGC-7901 GES1	miR-23b-3p NOTCH2	[34]
	Colorectal cancer (CRC)	Up	Proliferation Invasion EMT	SW480, HT29 LoVo, SW620 CaCO_2_, NCM460	–	[27]
	NSCLC	Up	Proliferation Invasion Migration Apoptosis	A549 NCI-H1299 PC9, LLC BEAS-2B	miR-30c-5p SOX9	[32]
2/5/6/7	Chronic lymphocytic leukemia (CLL)	Down	–	CD19 + B cells, CLL	–	[6]
1/2/6/7	HCC	Up	Replication Transcription	HepG2 HepG2.2.15 HepAD38 NTCP-HepG2	TRIM13 CCNB2 cccDNA	[36]
1/2/3/4/6/7	CC	Up	Proliferation Migration	HeLa, SiHa CaSki	ZFP36, P53 P21, NOTCH	[33]
3	Osteosarcoma	Up	Proliferation Migration	Saos-2, NHOst MG-63, 143B U-2OS	miR-337-3p JAK2	[25]
4/6	Pancreatic cancer (PC)	Up	Proliferation Invasion	AsPC-1 PaCa-2 HPDE6-C7	miR-455 SMAD2	[15]

### Clinical significance of DLEU2 expression

Abnormal expression of DLEU2 may provide new clues into the mechanism of tumor formation, indicating that DLEU2 may be a biomarker for cancer diagnosis. Interestingly, DLEU2 expression increases from stage I to IV and is almost absent in EC [12]. DLEU2 expression is significantly correlated with pathological grade and TNM stage in patients with GC [17]. DLEU2 is upregulated in osteosarcoma and its expression is proportional to tumor size and clinical stage [25]. The expression of DLEU2 in stage T III and IV was higher than that in stage II, and the high expression of DLEU2 was correlated with the later level of Stage N and prostate-specific antigen (TSA) [20]. In patients with LSCC, high expression of DLEU2 is associated with TNM and tumor stages. The expression of DLEU2 was higher in T3 and T4 than in T1 and T2. The expression of DLEU2 in stage III and IV was higher than that in stages I and II [23]. In addition, DLEU2 overexpression is closely associated with distant metastasis in CRC [27] and lymph node metastasis in patients with LSCC [23]. Furthermore, high DLEU2 expression is significantly associated with poor overall survival (OS) in EC [12], ccRCC [13], ESCA [23], PC [15], NSCLC [18], CRC [27], PCa [20], and LSCC [23]. To further study the prognostic effect of DLEU2, its prognostic value of DLEU2 in NSCLC tissue samples was verified based on its expression level, survival time, and survival status of DLEU2. The sensitivity, specificity, AUC, and cutoff value of DLEU2 in patients with NSCLC were 81%, 20.2%, 0.51, and 4.918, respectively [18]. The AUC and cutoff values for osteosarcoma were 0.8021 and 0.4566, respectively [25]. Accurate and early diagnosis is the key to prolonging patient survival. The abnormal expression of DLEU2 in specific tumors distinguishes pathological tissues from normal tissues, suggesting that DLEU2 may be a potentially useful diagnostic biomarker in cancer. Moreover, the expression of DLEU2 is closely related to the TNM stage and pathological grade and is mostly proportional to its expression in tumors. This indicated that DLEU2 is a promising prognostic biomarker (Table 2). However, the in vivo model undoubtedly focuses on demonstrating the reliability of DLEU2 as a biomarker for diagnosis and prognosis and studying its sensitivity and specificity, as well as the relationship between serum DLEU2 levels and tumor volume, recurrence, and metastasis.

**Table 2:** Clinical features of DLEU2 in tumors

Cancer type	Expression	Samples	Associated clinical features	Kaplan–Meier analysis	Cox regression analysis	References
Endometrial cancer (EC)	Up	50 Pairs of cancerous and normal tissues; female BALB/c nu mice	TNM stage	High DLEU2 expression was associated with poor overall survival (OS)	–	[12]
Prostate cancer (PCa)	Up	Male BALB/c nude mice	TNM stage, Gleason score prostate-specific antigen (PSA) level, residual tumor	The prognosis of prostate cancer patients with high expression of DLEU2 is poor	High DLEU2 expression was independently associated with a poor progression-free interval	[20]
Esophageal cancer (ESCA)	Up	–	–	High DLEU2 expression had significantly worse OS	DLEU2 exon 9 expression was an independent prognostic indicator of OS in cancer patients	[14]
Laryngeal squamous cell carcinoma (LSCC)	Up	66 Pairs of cancerous and normal tissues; female BALB/c nude mice	TNM stage, tumor stage lymph node metastasis	DLEU2 high expression had associations with poor prognosis	DLEU2 expression was an independent prognostic factor of LSCC	[23]
Non-small cell lung cancer (NSCLC)	Up	35 Pairs of cancerous and normal tissues; female BALB/c nu mice	–	High DLEU2 expression as well as lymph node metastasis was an independent prognostic factor of poor survival	–	[18]
		32 Pairs of cancerous and normal tissues; female BALB/c nu mice		DLEU2 was significantly associated with OS	–	[32]
Renal clear cell carcinoma (ccRCC)	Up	40 pairs of cancerous and normal tissues, 120 ccRCC cases; female BALB/c nu mice	–	DLEU2 was associated with poor prognosis	The expression of DLEU2 was negatively correlated with the survival rate of patients	[13]
Colorectal cancer (CRC)	Up	45 Pairs of cancerous and normal tissues	Distant metastasis	High DLEU2 expression levels exhibited poor OS and recurrence-free survival (RFS)	–	[27]
Osteosarcoma	Up	48 Pairs of cancerous and normal tissues; male BALB/c nude mice	Clinical stage tumor stage	–	–	[25]
Pancreatic cancer (PC)	Up	–	–	DLEU2 was associated with poor prognosis	–	[15]
Pediatric acute myeloid leukemia (AML)	Down	Non-leukemic group (*n* = 30)/pediatric AML (*n* = 28) (bone marrow samples)	–	DLEU2 was associated with poor prognosis	–	[8]
Gastric cancer (GC)	Up	75 Pairs of cancerous and normal tissues	Clinical stage TNM stage	–	–	[17]

## Function and regulatory mechanism of DLEU2 in human cancer

**Fig.2:**
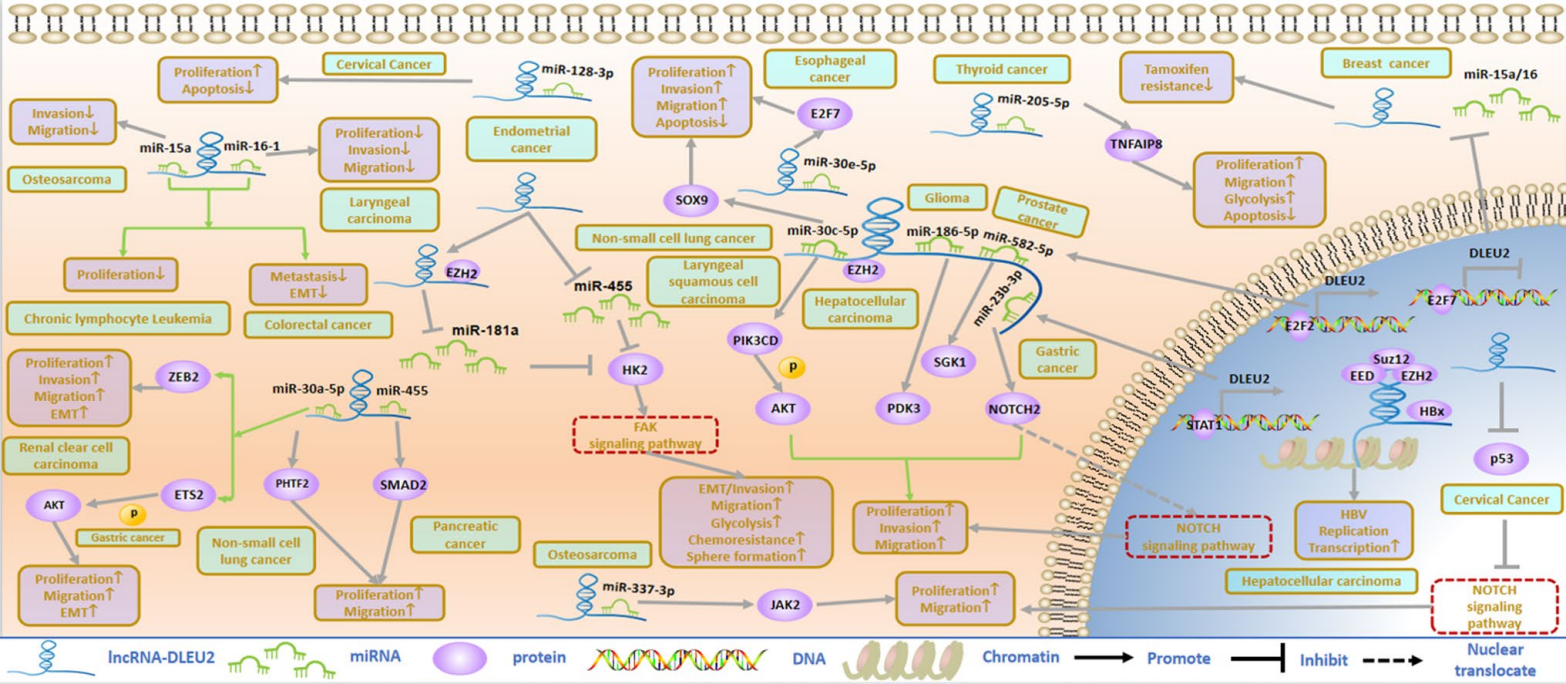
The functional mechanism of DLEU2 in tumors

The occurrence of tumors includes a series of events in which proliferation, migration, and invasion are the three characteristics of malignant tumors. DLEU2 is not only related to the above tumor characteristics, but also related to epithelial–mesenchymal transition (EMT) and glycolysis. This functional mechanism is explained in this section (Fig. 2).

### Proliferation and apoptosis

Studies have shown that the abnormal expression of DLEU2 is closely related to proliferation and apoptosis. Transfection of the DLEU2 plasmid in CLL resulted in rapid upregulation of miR-15a and miR-16-1, and overexpression of DLEU2 negatively regulated cyclin E1 and D1 in an miR-15a/miR-16-1 dependent manner, and inhibited proliferation and colony formation of tumor cells [29]. Compared with para-cancer tissues, the levels of DLEU2 and miR-16-1 in LC tissues are significantly decreased, and the upregulation of DLEU2 and miR-16-1 significantly reduces the ability of cells to proliferate [22]. In addition, DLEU2 promotes cancer cell proliferation by interacting with EZH2 in hepatocellular carcinoma (HCC) [30]. In PC, DLEU2 silencing inhibits SMAD2 by upregulating miR-455 and reducing tumor cell proliferation [15]. Members of the miR-30 family are associated with various tumors. DLEU2 negatively correlates with miR-30a-5p expression in NSCLC tissues and acts as a sponge for miR-30a-5p, reversing the tumor-promoting effects of DLEU2 by targeting PHTF2 [18]. In ESCA, DLEU2 promotes cell proliferation and reduces apoptosis by targeting miR-30e-5p and its downstream molecule E2F7 [31]. DLEU2 directly interacted with miR-30c-5p and targeted SOX9, thereby playing an active carcinogenic role in NSCLC. In vitro, DLEU2 knockdown inhibited cancer cell proliferation and induced apoptosis. It inhibits tumor growth and metastasis in vivo [32]. DLEU2, a competitive endogenous RNA, regulates the expression of PIK3CD by sponging miR-30c-5p and subsequently activates the Akt signaling pathway, promoting the proliferation of LSCC cells both in vivo and in vitro [23]. In CC, DLEU2 promotes cell proliferation and accelerates the cell cycle by targeting miR-128-3p [21] or by inhibiting the expression of p53 and the activity of the Notch signaling pathway [33]. In GC, DLEU2 is activated by STAT1 to inhibit miR-23b-3p and to promote NOTCH2 expression [34], thus promoting GC progression. In osteosarcoma, DLEU2 acts as a ceRNA to adsorb miR-337-3p, targeting JAK2, and promoting disease progression [25]. In gliomas, DLEU2 regulates PDK3 expression and glioma progression in an miR-186-5p dependent manner [16]. In addition, as a competitive endogenous RNA, DLEU2 may be activated by the transcription factor E2F2 to promote PCa progression by targeting the miR-582-5p/SGK1 axis [20]. Although the pathways by which DLEU2 promotes the function of malignant tumor cells are not consistent, most have one thing in common: the ceRNA mechanism. In the ceRNA mechanism, lncRNAs act as sponges for miRNAs and competitively bind to miRNA sites through their miRNA response elements to regulate mRNA expression [35]. The mechanism of action of DLEU2 and miRNAs may depend on the location of DLEU2 in the cancer cells. When lncRNAs are present in the cytoplasm, they generally act directly on downstream miRNAs. lncRNAs located in the nucleus are more likely to directly regulate and bind to downstream proteins. Currently, studies on the distribution of DLEU2 in cancer cells and its ability to directly bind to and regulate downstream proteins are limited. However, the ceRNA mechanism of DLEU2 may represent a therapeutic vulnerability. Because DLEU2 has multiple target miRNAs, adverse reactions based on lncRNA and miRNA interaction therapies should be evaluated.

### Migration, invasion, and EMT

In PC [15] and NSCLC [18], transwell assays showed that DLEU2 knockdown reduced the invasion ability of tumor cells. In CC, DLEU2 significantly downregulates p53 expression and inhibits the NOTCH signaling pathway, thus enhancing cell migration ability [33]. DLEU2 acts as an endogenous miRNA sponge for miR-337-3p, thereby promoting OS migration [25]. In HCC [30], LSCC [23], NSCLC [32], GC [34], glioma [16], ESCA [31] and PCa [20], transwell assay showed that the high expression of DLEU2 promoted the migration and invasion of tumor cells. In osteosarcoma [26] and LC [22], the expression of DLEU2 is contrary, and high expression of DLEU2 inhibits the migration and invasion of tumor cells. The migration and invasion of tumor cells are closely related to EMT. EMT is a marker of malignant tumor metastasis and an important biological process by which malignant tumors acquire the ability to migrate and invade. In CRC, AP4 directly inhibits the transcription of the miR-15a/16-1 host gene DLEU2, leading to the downregulation of DLEU2 and miR-15a/16-1 expression, thereby promoting EMT and tumor metastasis [28]. DLEU2 downregulation of miR-30a-5p promotes epithelial–mesenchymal transformation, migration, and invasion of ccRCC cells through 3'-UTR targeting ZEB2 [13]. DLEU2 promotes ETS2 expression and AKT phosphorylation in GC by binding to miR-30a-5p. Specifically, E-cadherin expression is upregulated in AGS and MKN-45 cells transfected with si-DLEU2, while the protein levels of N-cadherin, Vimentin, Snail, and Slug are downregulated [17]. Therefore, DLEU2 plays an important role in regulating EMT and eliminating intrinsic tumor targets can improve patient survival rates. However, the mechanism of action of DLEU2 in tumor EMT is not completely clear, and further research is needed.

### Other functions of DLEU2 in tumors

#### Glycolysis

In EC, DLEU2 plays a role in EC cells through two different mechanisms: DLEU2 induces HK2 expression by inhibiting miR-181a through competitive binding with miR-455 and interacting with EZH2. HK2 overexpression effectively promotes EMT and enhances aerobic glycolysis and chemical resistance in EC cells by activating FAK and downstream ERK1/2 signaling [12]. In TC, DLEU2 promotes aerobic glycolysis of thyroid cancer cells through the miR-205-5p/TNFAIP8 signaling axis [19]. Glycolysis is a characteristic feature of malignant tumors. In recent years, the inhibition of key enzymes in the glycolytic process of tumor cells has been explored as a target for tumor therapy. However, the energy metabolism of tumor cells is a complex process, and single-target therapy is insufficient to inhibit tumor development and can easily cause drug resistance. Therefore, multi-target combination therapy is worthy of attention, and according to the mechanism by which DLEU2 leads to glycolysis in tumors, combinations of DLEU2 and glycolysis-related proteins may be a new treatment plan.

#### Virus transcription and replication

In liver cancer caused by hepatitis B, HBX competes with EZH2 to bind to DLEU2, forming a complex that binds to the TRIMB/CCNB2 promoter, thereby promoting the replication and transcription of covalently closed circular DNA (cccDNA) and promoting cancer progression [36]. The biological functions of DLEU2 in malignancies are closely related to its downstream targets and signaling pathways. However, current studies on the downstream signaling pathways of DLEU2 are incomplete; therefore, the functional rule of DLEU2 through these signaling pathways cannot be obtained. The combination of DLEU2 targeting molecules with DLEU2 signaling pathway inhibitors may be another promising therapeutic strategy for tumors.

### Other regulatory mechanisms of DLEU2 in tumors

In pediatric AML, the DLEU2/Alt1 transcription start sites exhibit elevated methylation levels [8]. In addition, histone deacetylase inhibitors can acetylate the promoter of DLEU2, increasing its level of DLEU2 [37]. In addition, the promoter of DLEU2 was demethylated in PC and its expression was upregulated [38]. These studies suggest that the abnormal expression of DLEU2 may be related to epigenetic modifications, such as methylation and histone modification; however, the specific mechanism remains unclear. In addition, the combination of specific epigenetic characteristics with clinical indicators as well as the combination of epigenetically modified therapeutics with molecules holds promise for further advances in cancer diagnosis and treatment. In summary, DLEU2 is a promising biomarker for cancer diagnosis, prognosis, and treatment.

## Clinical application of DLEU2

### DLEU2 may be a promising new biomarker for the diagnosis of cancer

Recent studies have reported that DLEU2 is mostly associated with biological processes involved in the occurrence and development of tumors, including proliferation, apoptosis, invasion, metastasis, and EMT. However, reports on DLEU2 as a diagnostic marker for cancer are limited. DLEU2 has so far been examined mainly in various tumor tissues, but is also present in body fluids. Recently, a research team discovered that DLEU2 was derived from small serum extracellular vesicles (EV-DLEU2). Interestingly, in this validation cohort, compared to subjects with normal health (NL), chronic hepatitis (CH), and liver cirrhosis, EV-DLEU2 expression levels were significantly increased in all patients with the modified Union for International Cancer Control (mUICC) stage, suggesting that EV-DLEU2 can be used as a biomarker for the diagnosis of liver cancer. To further determine the value of EV-DLEU2 in the diagnosis of liver cancer, the diagnostic abilities of EV-DLEU2 and alpha-fetoprotein (AFP) were compared. The results showed that the AUC of AFP and EV-DLEU2 in mUICC stage I HCC were 0.508 and 0.885, respectively, compared with those in NL, CH, and liver cirrhosis, indicating that EV-DLEU2 performed significantly better than AFP in the detection of HCC at the very early stage. In addition, compared to AFP, EV-DLEU2 showed a higher negative rate in the CH and liver cirrhosis groups and a higher positive rate in the HCC group, suggesting that EV-DLEU2 has a good ability to distinguish between HCC and non-HCC patients. To determine the value of AFP and EV-DLEU2 in the diagnosis of early HCC, AFP, and EV-DLEU2 were tested at all stages of mUICC, with a positivity rate of up to 96% at mUICC stage I. The EV-DLEU2 group also showed a high positive rate (96%) in HCC with very early low AFP levels (≤ 20 ng·mL^− 1^) [39]. The abnormal expression of DLEU2 in various tumors indicates that DLEU2 is a useful diagnostic biomarker. However, whether there is abnormal expression of EV-DLEU2 in the body fluids of patients with other tumors requires further consideration and research on this issue necessary. Therefore, attention should be paid to the organ specificity of EV-DLEU2. Solving this problem may be a major breakthrough in clinical diagnosis. In summary, DLEU2 may be a promising biomarker for the early diagnosis of malignancies.

### Therapeutic value of DLEU2 in cancer

For decades, researchers have studied malignant tumors, but factors such as the easy recurrence and metastasis of malignant tumors and drug resistance have caused stumbling blocks in cancer treatment. Cancer remains one of the main causes of death in humans. The latest breakthrough in cancer research is the discovery and application of lncRNAs in cancer therapy [40]. Abnormal DLEU2 expression in cancer cells regulates the occurrence and development of tumors through a ceRNA mechanism [3], suggesting that DLEU2 is a potential therapeutic target. Reduced DLEU2 expression enhances drug resistance in EC [12] and BC [11]. In addition, DLEU2 promotes glycolysis in EC [12] and TC [19]; EMT in GC [17], CRC [27], and ccRCC [13]; and epigenetic changes in DLEU2 expression in PC [38] and AML [8], indicating the value of multi-target combination therapy with DLEU2.

## Conclusions and perspectives

DLEU2 is a potential biomarker and therapeutic target for various tumors. DLEU2 is mostly lost or downregulated in hematological diseases. In solid tumors, most are overexpressed oncogenes and a few are suppressor genes, which may be related to tissue specificity [21]. There are limitations to the research process. First, high or low expression of DLEU2 in the same tumor was speculated to be closely related to the cancer subtype classification and tumor heterogeneity. Second, there are seven different transcripts of DLEU2, but most studies have neglected the relationship between transcripts and downstream target molecules or tumor functions [7], which is another limitation.

DLEU2 mostly plays its role through the mechanism of ceRNA, which is also related to its distribution in cells. In most solid tumors, DLEU2 is mainly distributed in the cytoplasm and a small part in the nucleus. DLEU2 is distributed in the cytoplasm and exerts its effects primarily through miRNAs and their target molecules. It has been found that lncRNAs distributed in the nucleus can directly regulate and bind to downstream target proteins. For example, SNHG17 directly binds to and regulates NONO in the nuclei of SGC-7901 and AGS cells [41]. Only one previous study in the literature demonstrated that DLEU2 is mainly distributed in the nucleus [33]. Therefore, the role of DLEU2 in the nucleus warrants further investigation. The ceRNA mechanism of DLEU2 in the cytoplasm may represent a therapeutic vulnerability, but DLEU2 has many target miRNAs. Therefore, it is necessary to evaluate the adverse effects of lncRNA and miRNA interactions when it is used as a therapy.

Studies have reported that DLEU2 may be related to methylation and histone modification; however, the complete mechanistic pathway has not yet been elucidated. Based on the above literature, abnormal expression of DLEU2 in malignant tumors can promote the progression of multiple tumors. DLEU2 regulates biological processes, such as tumor proliferation, migration, and invasion, through miRNAs, proteins, or signaling pathways. This indicates the possibility of combining DLEU2 with molecular therapies. In addition, DLEU2 expression is closely associated with poor prognosis in patients with multiple tumors. Therefore, DLEU2 is a promising biomarker and therapeutic target for clinical applications. However, many of these findings have been established in tissues and cancer cell lines, and additional clinical validation in patients with cancer is needed to fully explore. Meanwhile, the sensitivity and specificity of DLEU2 in tumor diagnosis and prognosis and the correlation between serum DLEU2 and tumor recurrence and metastasis should be studied. The report of EV-DLEU2 in HCC has early diagnostic value; however, whether it also has EV-DLEU2 in other tumors and the specificity of the expression level of EV-DLEU2 in organs should be evaluated to fully explore the clinical value of DLEU2.

In addition, DLEU2 exhibits drug resistance in EC and BC, which provides a new direction for tumor treatment. However, research on DLEU2 in a variety of anticancer drugs needs to be supplemented in the future.

## Data Availability

All data generated or analyzed during this study are included in this published article.

## Abbreviations

DLEU2: Deleted in lymphocytic leukemia 2
lncRNAs: Long non-coding RNAs
ncRNAs: Non-coding RNAs
ceRNA: Competitive endogenous RNA
CLL: Chronic lymphocytic leukemia
B-CLL: B cell chronic lymphocytic leukemia
AML: Pediatric acute myeloid leukemia
PL: Pleomorphic liposarcoma
BC: Breast cancer
EC: Endometrial cancer
ccRCC: Clear cell renal cell carcinoma
ESCA: Esophageal cancer
PC: Pancreatic cancer
GC: Gastric cancer
NSCLC: Non-small cell lung cancer
TC: Thyroid cancer
PCa: Prostate cancer
LC: Laryngeal cancer
LSCC: Laryngeal squamous cell carcinoma
CC: Cervical cancer
CRC: Colorectal cancer
TSA: Prostate-specific antigen
OS: Poor overall survival
LAC: Lung adenocarcinoma
HCC: Hepatocellular carcinoma
EMT: Epithelial–mesenchymal transition
cccDNA: Covalently closed circular DNA
EV-DLEU2: Serum small extracellular vesicle-derived DLEU2
NL: Normal health
CH: Chronic hepatitis
mUICC: Modified Union for International Cancer Control
AFP: Alpha-fetoprotein
